# Process Optimization of In Situ Magnetic-Anisotropy Spark Plasma Sintering of M-Type-Based Barium Hexaferrite BaFe_12_O_19_

**DOI:** 10.3390/ma14102650

**Published:** 2021-05-18

**Authors:** Haetham G. Mohammed, Thar Mohammed Badri Albarody, Susilawati Susilawati, Scott Gohery, Aris Doyan, Saiful Prayogi, Muhammad Roil Bilad, Reza Alebrahim, Anwar Ameen Hezam Saeed

**Affiliations:** 1 Department of Mechanical Engineering, Universiti Teknologi Petronas, Seri Iskandar 32610, Malaysia; haetham_19000233@utp.edu.my; 2 Master of Science Education Program, University of Mataram, Mataram 83125, Indonesia; aris_doyan@unram.ac.id; 3 Physics Education, FKIP, University of Mataram, Mataram 83125, Indonesia; 4 Department of Mechanical Engineering, The University of Melbourne, Parkville, VIC 3010, Australia; scott.gohery@unimelb.edu.au; 5 Faculty of Applied Science and Enginering, Universitas Pendidikan Mandalika, Mataram 83126, Indonesia; saifulprayogi@ikipmataram.ac.id (S.P.); muhammadroilbilad@ikipmataram.ac.id (M.R.B.); 6 Industrial Engineering Department, University of Padova, 35131 Padova, Italy; reza.alebrahim@unipd.it; 7 Department of Chemical Engineering, Universiti Teknologi Petronas, Seri Iskandar 32610, Malaysia; anwar_17006829@utp.edu.my

**Keywords:** spark plasma sintering, sintering parameters, remanence, optimization, magnet, magnetic properties, anisotropic magnet

## Abstract

This paper introduces a new spark plasma sintering technique that is able to order crystalline anisotropy by in-series/in situ DC electric coupled magnetic field. The process control parameters have been investigated on the production of anisotropic BaFe_12_O_19_ magnets based on resulted remanence (Mr). Sintering holding time (H.T.), cooling rate (C.R.), pressure (P), and sintering temperature (S.T.) are optimized by Taguchi with L9 orthogonal array (OA). The remanent magnetization of nanocrystalline BaFe_12_O_19_ in parallel (Mr^ǁ^) and perpendicular (Mr^Ʇ^) to the applied magnetic field was regarded as a measure of performance. The Taguchi study calculated optimum process parameters, which significantly improved the sintering process based on the confirmation tests of BaFe_12_O_19_ anisotropy. The magnetic properties in terms of Mr^ǁ^ and Mr^Ʇ^ were greatly affected by sintering temperature and pressure according to ANOVA results. In addition, regression models were developed for predicting the Mr^ǁ^ as well as Mr^Ʇ^ respectively.

## 1. Introduction

Barium hexaferrite (BaFe_12_O_19_) has been one of the most widely used magnetic materials, accounting for nearly 90% of the $4 billion global market due to its superior properties such as low manufacturing costs, high Curie temperature, high coercivity, chemical stability, and corrosion resistance [1,2,3,4,5]. The production of hexagonal BaFe_12_O_19_ has reached 300,000 tons annually, equivalent to 50 g per person [2,6,7,8,9]. Due to its hexagonal structure, the material was called barium hexaferrite. The structure is magnetoplumbite/M-type with the general formula of MFe_12_O_19_ or MO _0.6_Fe_2_O_3_, in which M can be barium (Ba), strontium (Sr), or lead (Pb) [10].

In the last few decades, numerous techniques, such as the co-precipitation method [11], the sol-gel method [12], the hydrothermal/solvothermal method [13], and the solid-state method [14] have been developed to synthesize this barium-based hexaferrite BaFe_12_O_19_. Growing annealing temperature is frequently needed to obtain a pure phase of BaFe_12_O_19_, which deteriorates the magnetic properties. Barium hexaferrite single crystals have received a lot of attention in the last decade because of their high saturation magnetization and large magneto-crystalline anisotropy along the crystallographic c-axis [15], but due to their high-temperature melting point, synthesizing these single crystals is a very expensive and difficult task. A textured hexaferrite with high performance is seen as a good alternative since they have properties that are comparable to single-crystal hexaferrite [16].

In order to take advantage of the strong magnetic properties imparted by the magnetic uniaxial anisotropy, the M-type hexaferrite particles must be oriented along the c-axis of the crystal direction, which corresponds to a magnetically easy-axis and perpendicular to the hexagonal platelet-shaped plane [17].

Textured ceramics have been developed using a variety of processing techniques, including tape casting [18], multilayer screen printing [19], hot forging [20], and grain growth [21]. Synthesis of textured hexaferrite has previously been shown to be a time-consuming process that often necessitates special processing conditions such as a strong external magnetic field during the processing.

**Figure 1:**
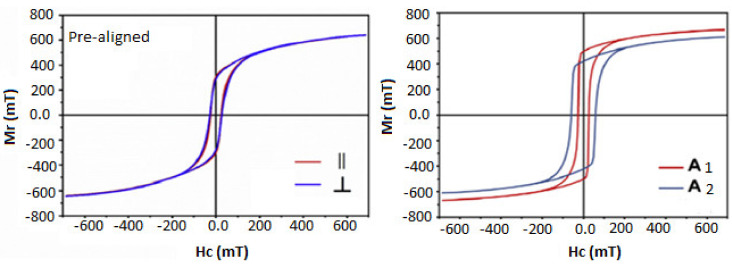
M^ǁ^ (H) curves for the in situ aligned samples without binder during the SPS process, adopted from Ref. [35].

In contrast to traditional sintering methods such as hot press (HP) or hot isostatic press (HP), spark plasma sintering (SPS) is a relatively new sintering process for the densification of various ceramic or metallic systems at a low sintering temperature and a very short sintering time [22,23,24,25,26,27]. SPS-compacted M-type barium-based hexaferrite bulk magnets have previously been published, but all samples showed only magnetically isotropic behavior [28,29,30,31]. Anisotropic barium hexaferrite is produced by adding NaCl salt during the sintering process at low sintering temperature. The addition of NaCl to the powder during the sintering process has proven to be an effective way in the alignment of particles along the magnetic easy-axis, but this method is limited to a certain low sintering temperature and also to a lengthy time-consumption [32]. So far, some permanent magnets coupled with SPS have been used in the alignment of sintered powders such as Sm-Co magnet with Curie temperature of 800 °C which was utilized during the sintering of Sm–Fe–N [33]; as well as Halbach magnet with a uniaxial magnetic field of 1T, which was used to assist spark plasma sintering of Co_80_Ni_20_ nanowires [34]; also two commercial Sm_2_Co_17_ bulk magnets (mµ0Br ≈1.10 T at 300 K, max working temperature (≈300 °C), which were utilized to assist anisotropic SPS-compacted MnBi magnets [35]. From the results obtained, the sintered magnets had high densities with anisotropic behavior and the external magnetic fields contributed to the enhancement of the Mr/Ms ratios of SPS-compacted samples, as in Figure 1.

The process parameters of spark plasma sintering, such as holding time, cooling rate, sintering temperature, pressure, and heating rate directly affect the mechanical, magnetic, and electrical properties of sintered materials. However, when the holding temperature is reached quickly during spark plasma sintering, compaction occurs with minimal grain growth. This phenomenon has been investigated using a variety of ceramic and metallic materials to create a full-densification nanostructure sample [25,36,37]. The sintering temperature increases in crystal size and density. In contrast, the heating rate had the opposite effect [38]. The density of sintered materials is affected by holding time, whereas, with the increase in holding time, the density of sintered boron carbide and Si_3_N_4_-SiC increased [39,40].

Indeed, the design of experiment (DOE) model emerged recently as a statistical method providing solutions for simplifying the manufacturing process of materials, optimizing process parameters, and minimizing unwanted trial by error approach. Therefore, the DOE approach helps to standardize production processes, reduce operational cost, minimize wastage, saves energy, saves time, and ensures accurate results in experimental studies [41]. In the 1920s, RA Fisher from England introduced the design of experiment (DOE) as a statistical tool used in studying the effect of multiple variables. However, the Taguchi method originated as the standard version of DOE by Dr. Genechi Taguchi, which was the most economically-friendly method used for experimental design [42]. Taguchi employs orthogonal arrays (OA), which are constructed so that the controllable variables can be analyzed with their respective responses at different levels in an ordered manner resulting in the smallest number of experiments, thus saving money and time. Limited studies have been reported in the literature in optimizing the spark plasma sintering. Moreover, Velmurugan et al. [43] used response surface methodology (RSM) to optimize SPS parameters such as temperature, holding time, and pressure for Si_3_N_4_-SiC/MgSiN_2_ composite. The optimized parameters enhanced the mechanical properties such as hardness and fracture toughness. Moreover, Ujah et al. [42] used Taguchi design in the experiment in their study on the optimization of SPS parameters of Al-CNTs-Nb nano-composite, where the sintering temperature, pressure, heating rate, and holding time were optimized. There was close agreement between DOE results (Taguchi predicted result) and experimental results (density and hardness).

As mentioned above, all previous researches focusing on the influence of the external magnetic field during the sintering process were restricted to permanent magnet usage with limited Curie temperature. There was also a lack of studies and analysis on the effect of various sintering parameters on the magnetic properties of soft and hard magnetic nanopowders during the sintering process.

This work introduces new spark plasma sintering (SPS) technique with magnetic anisotropy for magnetite ceramic sintering and grains alignment control to enhance the magnetic properties. Anisotropy of sintered barium ferrite BaFe_12_O_19_ is used to validate the SPS processing parameters that were optimized by (L9) Taguchi design of experiment (DOE) with (ANOVA) statistical tool. Furthermore, predictive magnetic remanence Mr^ǁ^ and Mr^Ʇ^ models were developed using regression analysis.

## 2. Materials and Methods

### 2.1. Materials

Nanocrystallites of M-type barium hexaferrite (BaFe_12_O_19_) powder with 100 nm average particle size (99% purity) was purchased from (Sigma-Aldrich (M) Sdn Bhd, Selangor D.E, Malaysia), and used as a starting material for this study.

### 2.2. Magnetic Anisotropic Spark Plasma Sintering (MASPS)

**Figure 2:**
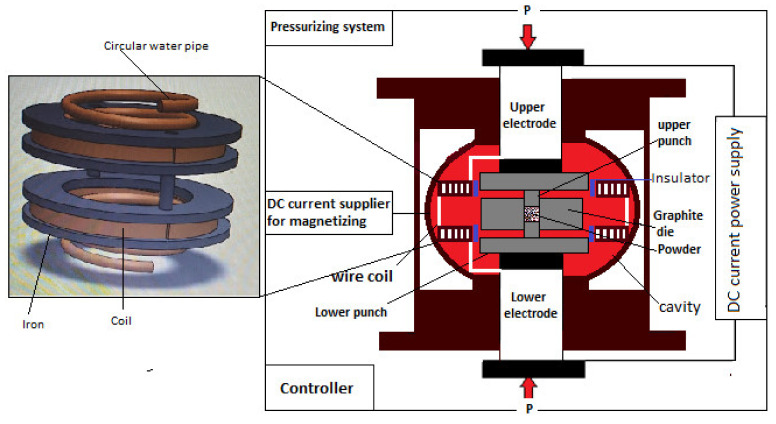
Schematic diagram of magnetic-anisotropic spark plasma sintering (MASPS).

A spiral magnetic coil with a rectangular cross-section, isolated by fiberglass and circular water pipes, is directly connected to the SPS’s cathode and anode (two spacers). This magnetic coil is designed to work coupled with SPS to generate a magnetic field in the same direction of electrical current, as shown in Figure 2. Table 1 presents the magnetic field generated from the coil at different sintering temperatures.

### 2.3. Sintering Parameters

The sintering process is mainly affected by different parameters. In this study, the effect of holding time (H.T.), cooling rate (C.R.), pressure (P), and sintering temperature (S.T.) on the magnetic properties of nanopowder are studied experimentally using a developed magnetically-assisted spark plasma sintering method. Table 2 shows the parameters and their levels used in this study, while the heating rate is used as a fixed parameter at 100 °C/min.

### 2.4. Design of Experiment (DOE)

Recently, DOE is considered as a statistical tool that employs the Taguchi orthogonal arrays (OA), so that the controllable variables can be analyzed with their respective responses at different levels in an ordered manner, which leads to the reduction of the number of experiments, thus saving money and time. However, by applying the Taguchi method, a system with three levels and four factors (parameters) L9 is the most suitable orthogonal array (OA) used as a DOE tool parameter in the experimental works.

A Minitab 17.0 (2017) software (Minitab LLC, State College, PA, USA) is used to input and analyze the sintering data. Based on the randomized OA, a total of 9 runs of experiments were conducted on MASPS in the order from trial 1 to trial 9. The experimental data for sintering process tests are shown in Table 3.

### 2.5. Experimental Process

BaFe_12_O_19_ powder was heated at 100 °C for 1 h to remove the moisture using a tube furnace under the Argon atmosphere. After the powder was cooled, it was taken out for weighing. Then, 0.4 ± 0.001 g of the powder was weighed using a digital balance. the powder was manually placed into a graphite mold, where the inner diameter of the graphite sleeve is 10 mm. The graphite mold consists of two 10 mm punches in diameter and a sleeve. The lower punch was inserted into the sleeve before the powder was poured into the sleeve. To avoid adhering to the interior of the mold and the punches, powders were separated by 0.1 mm graphite foils. The graphite mold with BaFe_12_O_19_ was then placed between two graphite spacers to compact the powder under specified pressures (DOE).

The sintering temperature, holding time, and cooling rate were specified by following the DOE, as shown in Table 4. Additionally, the surface temperature of the graphite sleeve was measured using IR thermometer sensor, where PID was utilized to monitor the sintering process. The heating rate is fixed at 100 °C/min. Manually, a gaussmeter was used to measure the generated magnetic field of the magnetic coil at the sintering temperature.

### 2.6. Sample Preparation and Measurement

**Figure 3:**
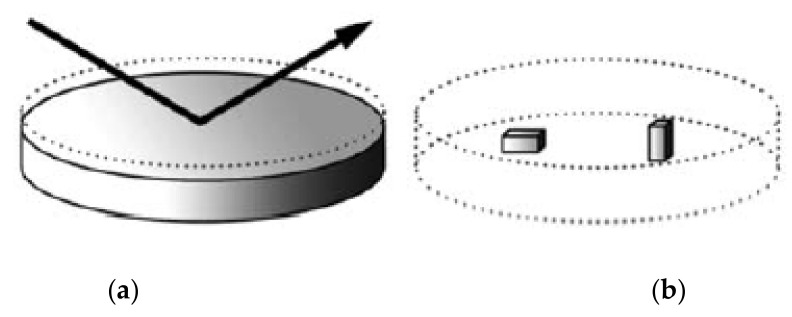
The scheme of (**a**) XRD of texture and (**b**) cut sample in orthogonal direction for measuring the remanent magnetization.

The sintered pellet of BaFe_12_O_19_ was removed from the graphite mold when the surface temperature of the graphite sleeve reached room temperature. Then, the grinder was utilized to remove all graphite foil from the sintered pellet. The next step was to characterize the crystallinity of the horizontal surface of sintered specimen for estimating the texture coefficient (TC) using X-ray diffraction (XRD, Bruker AXS D8 Advance, Karlsruhe, Ipoh, Malaysia). After that, the sintered specimen was vertically cut into two halves using the precious cutter to measure remanent magnetization for parallel (Mr^ǁ^) and perpendicular (Mr^Ʇ^) planes to the external magnetic field using vibrating sample magnetometer (VSM) (Lakshore model GMW 3474-140 Electromagnet, Westerville, Ipoh, Malaysia), as shown in Figure 3. Moreover, the applied magnetic field was 1 tesla.

### 2.7. S/N Ratio Analysis

In this part, the optimal level for each response is calculated using Taguchi’s S/N ratio analysis. For the response, such Mr^ǁ^ with the-larger-the-better (LTB) quality characteristic is chosen, as in Equation (1). In contrast, for Mr^Ʇ^, the-smaller-the-better quality characteristic is shown in Equation (2).

(1) $$\eta_{i}=-10\;log_{10}\frac{1}{N}\overset{r}{\underset{i=1}{\sum }}\left(\frac{1}{{y_{i}}^{2}}\right)\;\;\;\;i=1,\;2,\;\ldots \;r,\;\;\;\;\;\;\;$$

(2) $$\eta_{i}=-10\;log_{10}\frac{1}{N}\overset{r}{\underset{i=1}{\sum }}\left({y_{i}}^{2}\right)\;\;\;\;i=1,\;2,\;\ldots \;r,\;\;\;\;\;$$

where *N* is the number of tests, ${y_{i}}^{2}$ is the output, and $\eta_{i}$ is the S/N ratio.

### 2.8. Validation

To validate the optimized parameters of the S/N ratio, the sintering of BaFe_12_O_19_ powder was conducted based on the optimized parameters. Then, all the previous steps were repeated to measure and characterize the remanent magnetization (Mr^ǁ^ and Mr^Ʇ^) and XDR, respectively. After that, Minitab 17.0 software was used to analyze linear regression and construct predictive mathematical models for the dependent variables Mr^ǁ^ and Mr^Ʇ^ as a function of holding time, cooling rate, pressure, and sintering temperature, respectively, in the current study. The collected results from predictive mathematical models were compared with the experimental results of optimized parameters.

## 3. Results and Discussion

### 3.1. Taguchi Method

A loss function is used by Taguchi, which converts the difference in experimental values and targets into an S/N ratio, which is a ratio of mean to the standard deviation. Taguchi uses signal and noise to represent the wanted and unwanted values for the response. S/N ratio has been divided into three groups: the-medium-the-better, the-larger-the-better, and the-lower-the-better based on the response requirements. In this study, the characterized quality of the responses like Mr^ǁ^ and Mr^Ʇ^ are the-larger-the-better and the-lower-the-better, respectively. Therefore, Equations (1) and (2) have been used to calculate the S/N ratio, as shown in Table 4 [44].

### 3.2. The Influence of Process Parameters on (Mr^ǁ^) and (Mr^Ʇ^)

**Figure 4:**
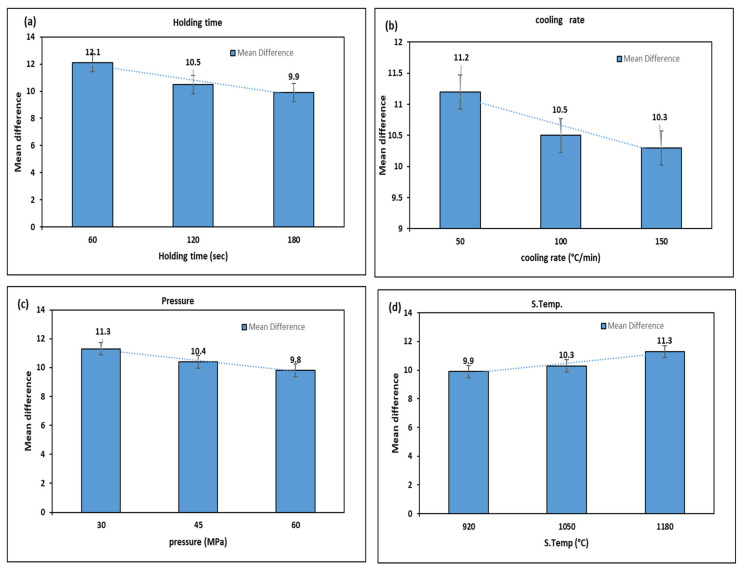
The effect of sintering parameters on mean difference between Mr^ǁ^ and Mr^Ʇ^: (**a**) Holding time; (**b**) Cooling rate; (**c**) Pressure; (**d**) S. temp.

The effect of sintering parameters on Mr^ǁ^ and Mr^Ʇ^ of sintered M-type barium hexaferrite nanopowder is presented in Figure 4a–d. Where the larger the mean difference between the Mr^ǁ^ and Mr^Ʇ^, the better is the anisotropic behavior of the sintered powder. It is observed from Figure 4a that the mean difference between Mr^ǁ^ and Mr^Ʇ^ is decreased when holding time is increased. This dramatical dropping in the mean difference from 12.10 to 9.90 when the holding time increased from 60 to 180 s is caused by the dropping in the magnetic properties of the materials, where the increase in holding time leads to the decrease in the grain size which causes a reduction in the remanence magnetization of the sintered materials [35,38].

The cooling rate has an impact on the mean difference between Mr^ǁ^ and Mr^Ʇ^, where the lower cooling rate results in a high mean difference. Therefore, when the value of the cooling rate is 50 (°C/min) the mean difference is 11.20, which is greater than the values of cooling rate at 100 and 150 (°C/min). This can be observed from Figure 4b where the mean differences of 100 and 150 (°C/min) are 10.5 and 10.30, respectively. The reason is that the lower cooling rate gives sufficient time for the external magnetic field to align the grains and prevent the crack to be appeared in the sintered specimen. In the previous studies, it was found that better alignment occurred at a lower cooling rate [45].

The pressure greatly influences the grains’ alignment, but the low pressure exerted on the powder requires little force to rotate the grain. As the pressure increases, the external magnetic field required to align the grains increases. Furthermore, the high pressure leads to align the hexagonal crystals in the perpendicular direction to the pressure (magnetic field) [46]. As shown in Figure 4c, the mean difference is decreased from 11.30 to 10.50 when the pressure is increased from 30 to 45 MPa, respectively. It can be also observed that the mean difference is decreased to 9.80 as the pressure is further increased to 60 MPa.

As commonly known, the temperature is generated due to the increase in the electrical current that affects the reorientation of grains in the same direction of magnetic flux. Moreover, the external magnetic field and the electrical current are in proportion. In other words, the increase in electrical current leads to a rise in the temperature and external magnetic flux together. Those two factors play an important role to reorient the grains. It is important to note that the increase in temperature will increase the activation energy of particles that will assist the reorientation of grains due to the external magnetic flux [47]. In contrast to the effect of the sintering parameters mentioned above, the sintering temperature shown in Figure 4d is directly proportional to the mean difference between Mr^ǁ^ and Mr^Ʇ^ in the sense that when the temperature was 920 °C, the mean difference was 9.90. Furthermore, the increment in sintering temperature from 1050 to 1180 °C caused an increase in the mean difference of 10.30 to 11.30, respectively. This effect explained that the increment in the external magnetic field, which resulted from the high current used in rising the sintering temperature, caused a proper alignment in the grains to the easy-axis of crystalline. Furthermore, the sintering temperature caused grain growth in the c-axis which played a secondary role in increasing the remanent magnetization of the sintered sample [48].

### 3.3. The Optimum Selected Parameters for Mr^ǁ^ and Mr^Ʇ^

**Figure 5:**
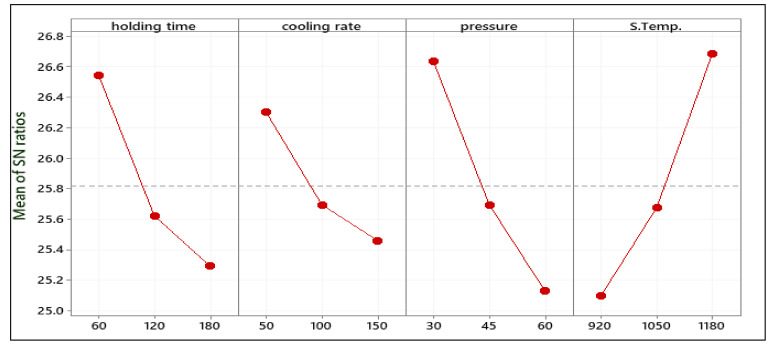
Mean S/N ratio of Mr^ǁ^.

The response table for the gained S/N ratio of Mr^ǁ^ is displayed in Table 5. The mean S/N ratio graph obtained using the Minitab software tool is shown in Figure 5. The minimal variance difference between the expected output and the measured output is represented by a higher S/N ratio. From Figure 5, it may be observed that the highest mean S/N ratio obtained for Mr^ǁ^ occurred at 60 s holding time, 50 °C/min cooling rate, 30 MPa pressure, and 1180 °C sintering temperature. H.T. = 60 s, C.R. = 50 °C/min, P = 30 MPa, and ST 1180 °C were the predicted optimum process parameters for obtaining high remanent Mr^ǁ^ in parallel to the magnetic field using the Taguchi method. The predicted combination of the optimum set was represented as H.T._1_-C.R._1_ -P_1_ -S.T._3_ for Mr^ǁ^.

**Figure 6:**
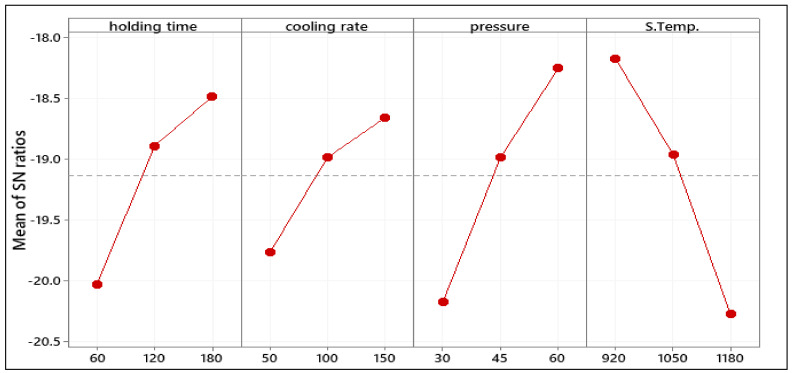
Mean S/N ratio of Mr^Ʇ^.

The means of the S/N ratio response table for Mr^Ʇ^ are presented in Table 6. H.T. = 60 s, C.R. = 50 °C/min, P = 30 MPa, and S.T. = 1180 °C were the predictable optimum process parameters for obtaining the low Mr^Ʇ^ as in Figure 6. For Mr^Ʇ^, the predicted optimum combination was H.T._1_–C.R._1_ -P_1_ -S.T._3_.

### 3.4. Confirmation Test

Conformational tests must be performed to validate the optimal conditions predicted by Taguchi. The response was estimated and verified using the predicted S/N ratio under predicted optimal sintering conditions. The confirmation experiments were carried out at the Taguchi predicted optimum sintering parameters, and the results are shown in Table 7 and Table 8 for Mr^ǁ^ and Mr^Ʇ^, respectively. The predicted optimum sintering parameters for both Mr^ǁ^ and Mr^Ʇ^ give an enhancement in the performance representative results. Table 7 and Table 8 show that for both Mr^ǁ^ and Mr^Ʇ^, the S/N ratios of predicted and optimal sintering processes are very close. The improved S/N ratios for Mr^ǁ^ and Mr^Ʇ^ were found at the optimum sintering parameters with 5.23 dB and 6.09 dB values, respectively, compared to the initial settings. Table 7 and Table 8 display the confirmation results of Mr^ǁ^ and Mr^Ʇ^. The optimum predicted parameters given by Taguchi provide better results than the initial parameter conditions according to the confirmation experiments. Hence, the increment in Mr^ǁ^ and the reduction in Mr^Ʇ^ were 26.56% and 27.83%, respectively.

### 3.5. XRD Analysis

**Figure 7:**
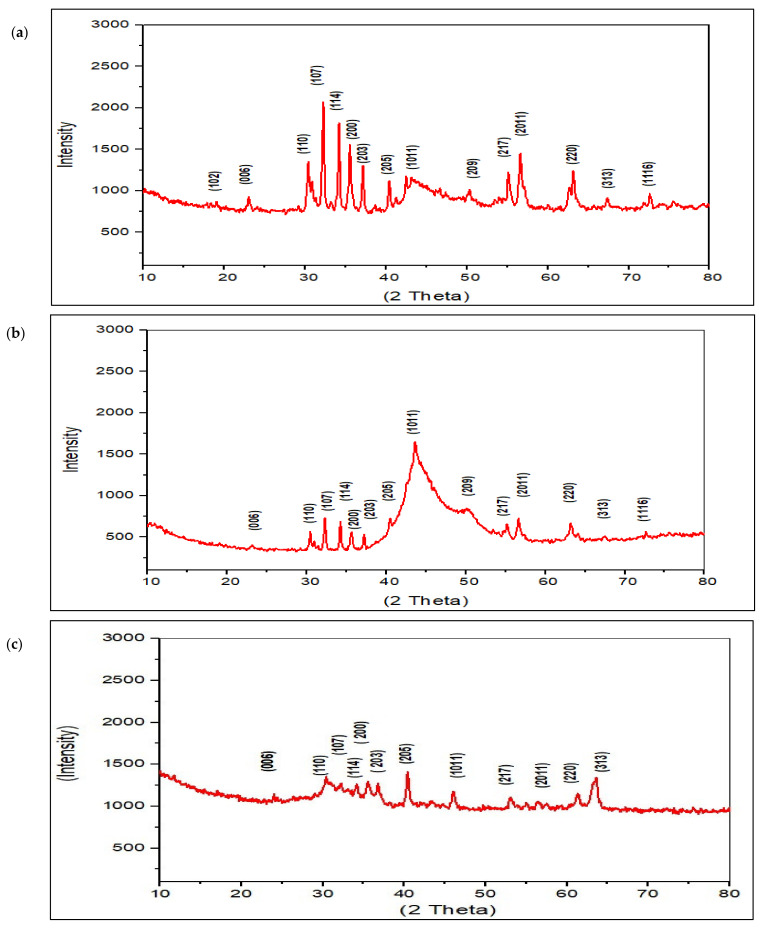
XRD of (**a**) Sintered sample at the optimized parameters; (**b**) Sintered sample at the initial parameters, and (**c**) Starting powder used in this study.

The grain orientation and phase analyses were conducted using an XRD system. The relative intensity ratio between (006) and (114) peaks (I_006_/I_114_) was determined for the samples sintered at the initial sintering parameters and at the optimized sintering parameters taken from Taguchi result as shown in Figure 7a–c, where the increase in the relative intensity means that the grains lead to be reoriented along the c-axis (highly textured) [49]. Accordingly, the orientation of the grain can be obtained qualitatively by the Texture Coefficient (TC) [50]. TC value can be calculated as in Equation (3) [51]:

(3) $$TC_{\left(hkl\right)}=\frac{I_{\left(hkl\right)}/I_{0(hkl)}}{(\frac{1}{N})\left[\sum_{N}\frac{I_{\left(hkl\right)}}{I_{0(hkl)}}\right]}$$

where, $I_{0(hkl)}$ , and $I_{\left(hkl\right)}$ represent the standard and the measured relative intensities of the specific crystal plans (hkl), and N represents the number of peaks. The calculated TC values are displayed in Table 9.

The relative intensity ratio (I_006_/I_114_) of the sintered sample at the initial process parameters is 40.60, and this value was improved for the sample with the optimized parameters to 51.23. This increase in the relative intensity demonstrated that the grains are oriented along the c-axis. The broadening occurs, as seen in Figure 7c, in between 40 and 50 of 2Ɵ indicates the grain refinement and high lattice strain [52]. The amorphization which can be observed from the decrease in the intensities of the peak has resulted from the graphite foil used during the sintering, where the increase in the intensity of the peak (1011) matched the highest peak intensity of the graphite.

These findings were also proved by the TC. Table 9 clearly shows that the (006) plane of the two samples have the highest TC values, indicating that all of the samples have a c-axis desired orientation. It is important to note that the TC value of plane (006) of the sample sintered at the optimized parameters is higher than that of the initial parameters. In contrast, the TC value of the plane (114) decreased from 2.0 to 1.70 of the samples sintered at the initial and optimized parameters, respectively. Thus, this confirms that the preferred growth orientation of BaFe_12_ O_19_ is along the c-axis.

## 4. ANOVA Analysis

ANOVA identifies the process variable that has the greatest influence on the output. Table 10 and Table 11 show the ANOVA results for Mr^ǁ^ and Mr^Ʇ^, respectively. According to Table 10, sintering temperature has the greatest influence on Mr^ǁ^, followed by pressure, holding time, and cooling rate. Sintering temperature, pressure, holding time, and cooling rate contributed to Mr^ǁ^ by 35.25%, 31.48%, 22.95%, and 10.32%, correspondingly, as indicated in Table 10. Similarly, Mr^Ʇ^ was typically influenced by sintering temperature, pressure, holding time, and cooling rate. The respective percentages contribution of sintering temperature followed by pressure, holding time, and cooling rate were 36.99%, 31.02%, 21.24%, and 10.74%, respectively, as shown in Table 11. Due to the magnetic field being influenced by the current used for heat generation, the ANOVA study revealed that both Mr^ǁ^ and Mr^Ʇ^ were significantly affected by the sintering temperature.

## 5. Modeling

The Equations (4) and (5) were driven from the linear regression to predict the values for Mr^ǁ^ and Mr^Ʇ^, respectively.

(4) $${\mathrm{Mr}}^{ǀ}=15.75-0.02261\;H.T.-0.01920\;C.R.-0.669\;P\;+0.01295\;S.T.$$

(R2 = 95.59%)

(5) $${\mathrm{Mr}}^{Ʇ}=6.62-0.01269\;H.T.-0.01170\;C.R.-0.3940\;P+0.00781\;S.T.$$

(R2 = 95.74%)

**Figure 8:**
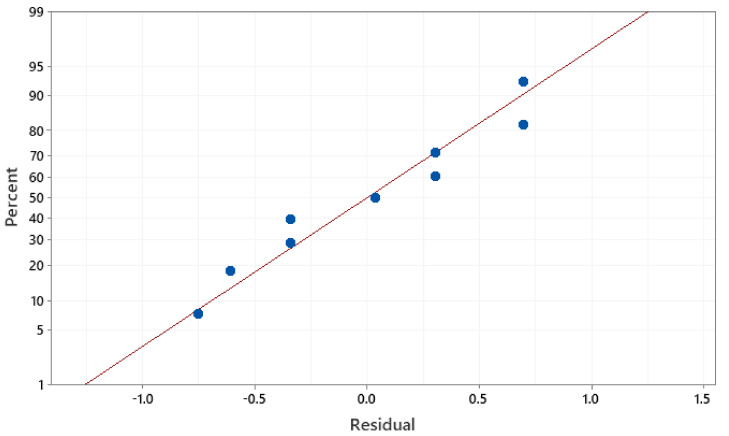
Normal probability plot of the residuals for Mr^ǁ^.

**Figure 9:**
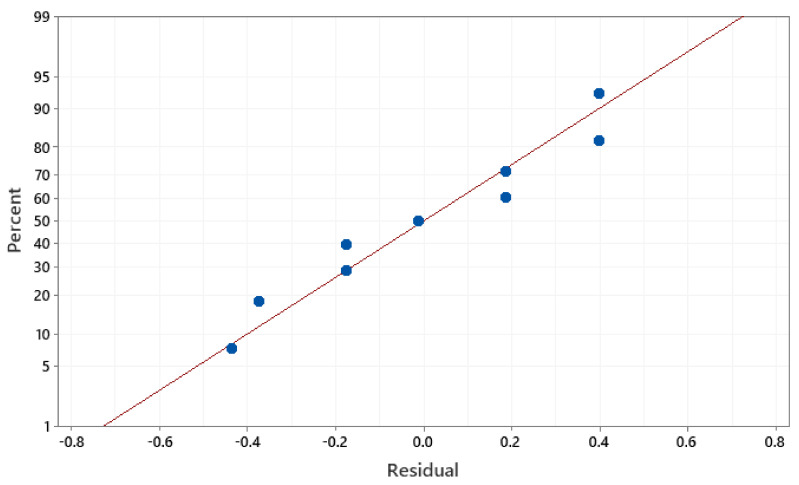
Normal probability plot of the residuals for Mr^Ʇ^.

The developed regression models of Mr^ǁ^ and Mr^Ʇ^ have a high coefficient of determination R^2^ values of 95.59% and 95.74%. This indicates that the dependent and independent variables in the established model are well-matched. The significance of the coefficient in the forecast model was validated by the residual plot. In contrast, the model’s error is considered normally distributed and significant when the plot is in a straight line. As observed from Figure 8 and Figure 9, the residual of the Mr^ǁ^ and Mr^Ʇ^ are near a straight line, implying that the developed models are significant.

Confirmation tests were used to validate the built models, and the tested results were selected randomly from the configuration of the experimental matrix. The results verified that the predicted and experimental values were in good agreement with the parameters described in Table 12.

**Figure 10:**
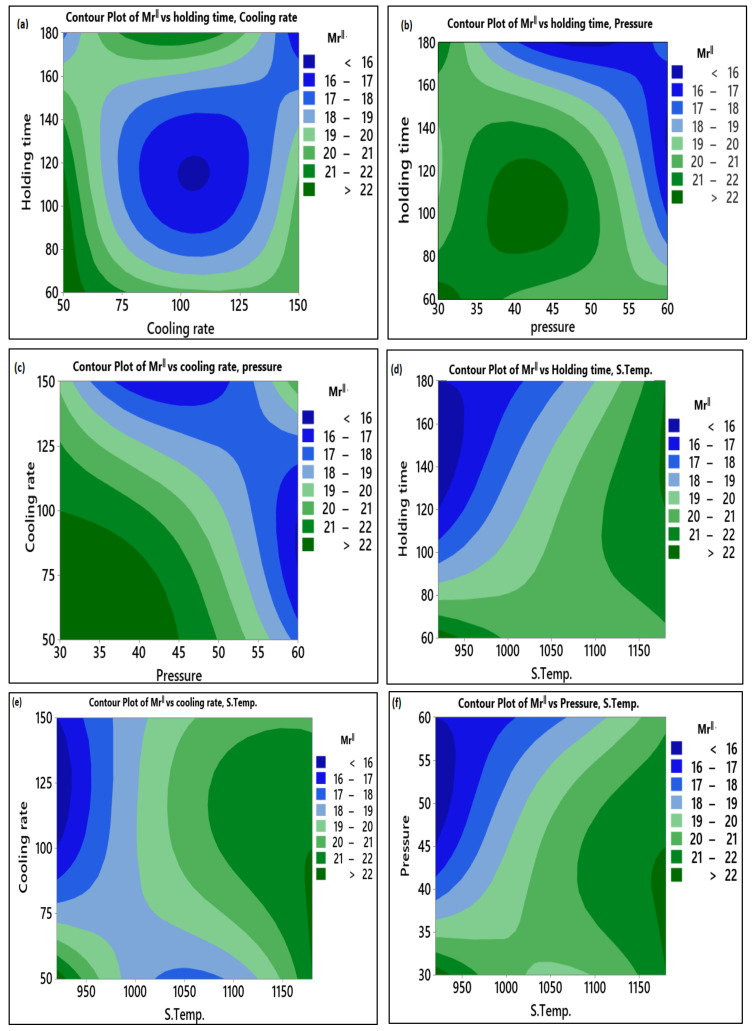
Contour plot for Mr^ǁ^: (**a**) Holding time vs. cooling rate; (**b**) Holding time vs. pressure; (**c**) Cooling rate vs. pressure; (**d**) Holding time vs. S. temp.; (**e**) Cooling rate vs. S. temp.; (**f**) Pressure vs. S. temp.

Contour plots can analyze the relationship between the response and two variables by viewing distinct contours of the expected response variables. The contour plots shown in Figure 10 represent the relation between the sintering parameters and the remanent magnetization in the parallel axis with external magnetic field Mr^ǁ^ values. Figure 10a shows that the low level of holding time and cooling rate generates a high Mr^ǁ^ value. Figure 10b indicates that high Mr^ǁ^ could be attained when the pressure ranged between 40 and 45 MPa and holding time between 100 and 120 s. It was observed in Figure 10c that Mr^ǁ^ is maximum when the cooling rate and pressure ranged between 50 and 100 °C/min and 30–35 MPa, respectively. High sintering temperature with high holding time leads to high Mr^ǁ^ as in Figure 10d. With high sintering temperature and cooling rate in between 75 and 100 s, the Mr^ǁ^ increases, as in Figure 10e.

**Figure 11:**
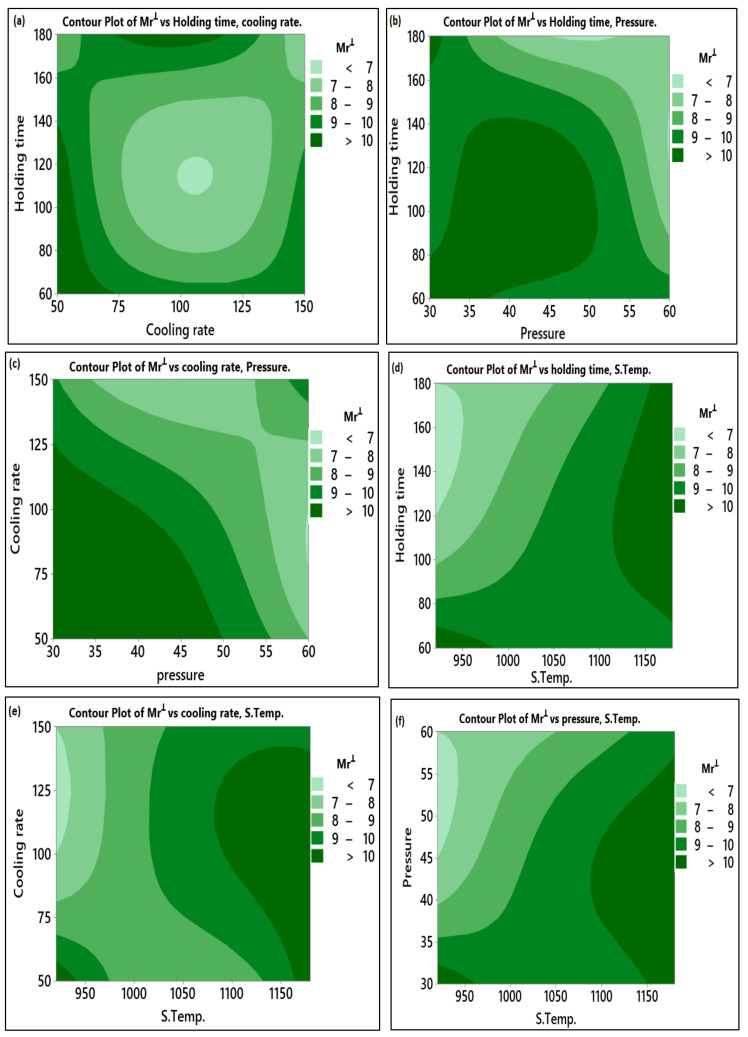
Contour plot for Mr^Ʇ^: (**a**) Holding time vs. cooling rate; (**b**) Holding time vs. pressure; (**c**) Cooling rate vs. pressure; (**d**) Holding time vs. S. temp.; (**e**) Cooling rate vs. S. temp.; (**f**) Pressure vs. S. temp.

Similarly, in Figure 10f, the high value of Mr^ǁ^ was obtained at high sintering temperature and pressure ranged between 35 and 40 MPa. The relation between the Mr^Ʇ^ and the variables is presented in Figure 11. From the observed data, the sintering temperature contributed to the increase of the remanent magnetization of the whole sample from different directions. The remanent magnetization in the perpendicular direction increased with the increase in samples’ overall remanent of about 50% of the Mr^ǁ^. The other parameters have the same effect on the Mr^Ʇ^ and Mr^ǁ^.

## 6. Assessment of Mr^ǁ^ and Mr^Ʇ^ for Sintered BaFe_12_O_19_

Mr^ǁ^ and Mr^Ʇ^ of sintered BaFe_12_O_19_ by using different sintering technology and MASPS were compared and listed in Table 13. The Mr^ǁ^ and Mr^Ʇ^ values of SPS with NaCl are found to be 29.30 and 24.90, respectively. On the other hand, the Mr^ǁ^ and Mr^Ʇ^ values of MASPS are 27.10 and 7.70, respectively, which shows that SPS with NaCl resulted with higher values of Mr^ǁ^ and Mr^Ʇ^. This comparison does not evidentially represent the better anisotropic behavior of the sintered sample by SPS with NaCl, rather the anisotropic behavior is represented by the relative ratio between Mr^ǁ^ and Mr^Ʇ^. In that sense, the relative ratio of the sintered sample by MASPS is 71.61%, which is higher than that of the SPS with NaCl with a value of 26%. This analysis shows that the anisotropic behavior of MASPS is better than that of SPS with NaCl. The high Mr^ǁ^ and Mr^Ʇ^ values of SPS with NaCl can be justified by the presence of high grain-size powder.

## 7. Conclusions

Anisotropic BaFe_12_O_19_ magnets were achieved by a developed magnetic-anisotropy spark plasma sintering at the low magnetic field coupled with sintering current. From the analyzed results, the following conclusions were drawn:

- The optimum setting sintering parameters for obtaining the high Mr^ǁ^ was found as H.T. = 60 s, C.R. = 50 °C/min, P = 30 MPa and S.T. = 1180 °C ((H.T.)_1_-(C.R.)_1_-P_1_-(S.T.)_3_) using Taguchi method. It was observed that a 26.57% increment of Mr^ǁ^ was found at the Taguchi determined optimum sintering condition.
- The optimum sintering combination for Mr^Ʇ^ determined by the Taguchi method is the same as the combination for obtaining Mr^ǁ^. In contrast, the increment in the remanence of the sintered sample in the parallel direction leads to a decrease in the remanence of the perpendicular direction in about 50% of Mr^ǁ^. In the Taguchi optimized sintering condition, the amount of reduction in the Mr^Ʇ^ was 27.83%.
- It was observed from the ANOVA analysis that Mr^ǁ^ and Mr^Ʇ^ were significantly influenced by the sintering temperature with a contribution of 35.25% and 36.99%, respectively, followed by pressure, holding time, and cooling rate.
- Based on the well-founded optimal sintering parameters, it can be suggested that MASPS with a high magnetic field could be a promising approach to achieve anisotropic permanent magnets because both Mr^ǁ^ and Mr^Ʇ^ can be tailored to reach the desired properties.
- From the XRD, the improvement in the relative intensity ratio between (006) and (114) peaks (I*_006_*/I*_114_*) from 40.60 to 51.29 for the sintered powder at the initial and optimized process parameters proved that the grains have been oriented along the c-axis, which was also supported by the TC values of the plane.
- From the developed mathematical models Mr^ǁ^ and Mr^Ʇ^, a close agreement between the predicted results and experimental results was observed. Hence, the developed models could correct sintering parameters for producing anisotropic magnets without conducting trial experiments.
- Further studies are recommended to investigate the effect of process parameters of MASPS on the mechanical and microstructure behavior of the sintered nanopowder.

## Figures and Tables

**Table 1:** Magnetic field (mT) to current (A) relation.

NO	Current (A)	Temperature (°C)	Magnetic Field (mT)
1	150	650	40
2	200	780	44
3	250	920	70
4	300	1050	80
5	350	1180	90

**Table 2:** Sintering process parameters and their levels.

Symbols	Process Parameters	Unit	Levels		
			1	2	3
H.T.	Holding time	S	60	120	180
C.R.	Cooling rate	°C/min	50	150	250
P	Pressure	MPa	30	45	60
S.T.	Sintering temperature	°C	920	1050	1180

**Table 3:** Experimental data for the sintering process.

Run	Holding Time (min)	Cooling Rate (°C/min)	Pressure (MPa)	Sintering Temperature (°C)
1	60	50	30	920
2	60	100	45	1050
3	60	150	60	1180
4	120	50	45	1180
5	120	100	60	920
6	120	150	30	1050
7	180	50	60	1050
8	180	100	30	1180
9	180	150	45	920

**Table 4:** The results of experiments and the calculated S/N ratio.

Exp. Runs	Results		S/N Ratio of Results	
	Mr^ǁ^ (emu/g)	Mr^Ʇ^ (emu/g)	Mr^ǁ^ (dB)	Mr^Ʇ^ (dB)
1	22.70	10.89	27.12	−20.74
2	20.30	9.50	26.14	−19.55
3	20.80	9.78	26.36	−19.80
4	22.00	10.60	26.84	−20.50
5	16.00	7.00	24.08	−16.90
6	19.80	9.20	25.93	−19.27
7	17.66	8.00	24.93	−18.06
8	22.00	10.60	26.84	−20.50
9	16.00	7.00	24.08	−16.90

**Table 5:** Mean S/N ratio response table for Mr^ǁ^.

Symbol	Process Parameters	Mean S/N Ratio				
		Level 1	Level 2	Level 3	Max–Min	Rank
H.T.	Holding time (s)	**26.54**	25.62	25.29	1.25	3
C.R.	Cooling rate (A/min)	**26.30**	25.69	25.46	0.84	4
P	Pressure (KN)	**26.63**	25.69	25.13	1.51	2
S.T.	S. temp. (°C)	25.10	25.67	**26.69**	1.59	1

Larger—better.

**Table 6:** Mean S/N ratio response table for Mr^Ʇ^.

Symbol	Process Parameters	Mean S/N Ratio				
		Level 1	Level 2	Level 3	Max–Min	Rank
H.T.	Holding time (s)	**−20.03**	−18.89	−18.49	1.54	3
C.R.	Cooling rate (A/min)	**−19.77**	−18.99	−18.66	1.11	4
P	Pressure (KN)	**−20.17**	−18.99	−18.26	1.92	2
S.T.	S. temp. (°C)	−18.18	−18.96	**−20.27**	2.09	1

Smaller—better.

**Table 7:** Confirmation test results for Mr^ǁ^.

	Optimal Process Parameters		
	Initial Process Parameters	Prediction	Experimental
Levels	(H.T.)_2_-(C.R.)_2_-P_2_-(S.T.)_2_	(H.T.)_1_-(C.R.)_1_-P_1_-(S.T.)_3_	(H.T.)_1_-(C.R.)_1_-P_1_-(S.T.)_3_
Mr^ǁ^	19.91	28.71	27.11
S/N ratio (dB)	24.84		29.72
Improvement in S/N ratio (dB)	5.23		
Percentage of the increment in Mr^ǁ^ (emu/g)	26.56%		

**Table 8:** Confirmation test results for Mr^Ʇ^.

	Optimal Process Parameters		
	Initial Process Parameters	Prediction	Experimental
Levels	(H.T.)_2_-(C.R.)_2_-P_2_-(S.T.)_2_	(H.T.)_1_-(C.R.)_1_-P_1_-(S.T.)_3_	(H.T.)_1_-(C.R.)_1_-P_1_-(S.T.)_3_
Mr^Ʇ.^	9.87	−22.83	7.72
S/N ratio (dB)	−19.91		−26.01
Improvement in S/N ratio (dB)	6.09		
Percentage of the reduction in Mr^Ʇ^ (emu/g)	27.83%		

**Table 9:** Texture coefficient (TC).

Sample	Crystal Plane					
	(006)	(107)	(114)	(1011)	(217)	(313)
Intial process parameters	11.90	3.50	2.00	6.40	2.00	1.20
Optimized parameters	12.20	4.40	1.70	5.90	1.50	0.80

**Table 10:** ANOVA for Mr^ǁ^.

Source	Degree of Freedom	Sum of Square	Means Square	% Contributions
H.T. (s)	2	2.53	1.26	22.95
C.R. (A/min)	2	1.13	0.56	10.32
P (MPa)	2	3.47	1.73	31.48
S.T. (°C)	2	3.89	1.94	35.25
Total	8	11.03		100

**Table 11:** ANOVA FOR Mr^Ʇ^.

Source	Degree of Freedom	Sum of Square	Means Square	% Contributions
H.T. (s)	2	3.84	1.92	21.24
C.R. (A/min)	2	1.94	0.97	10.74
P (MPa)	2	5.62	2.80	31.02
S.T. (oC)	2	6.70	3.35	36.99
Total	8	18.11		100

**Table 12:** The confirmed results for the developed model.

Run	Experimental		Predicted		Residuals		% Error	
	Mr^ǁ^ (emu/g)	Mr^Ʇ^ (emu/g)	Mr^ǁ^ (emu/g)	Mr^Ʇ^ (emu/g)	Mr^ǁ^ (emu/g)	Mr^Ʇ^ (emu/g)	Mr^ǁ^ (emu/g)	Mr^Ʇ^ (emu/g)
2	20.30	9.50	19.69	9.17	−0.60	−0.32	2.97	3.44
5	16	7	16.35	7.18	0.355	0.18	2.21	2.57
8	22	10.6	23.05	11.17	1.05	0.57	4.78	5.40
9	16	7	15.70	6.81	−0.29	−0.18	1.81	2.58

**Table 13:** Mr^ǁ^ and Mr^Ʇ^ values of sintered BaFe_12_O_19_ with different methods.

Method	Mr^ǁ^ (emu/g)	Mr^Ʇ^ (emu/g)	References
Magnetic-field-assisted hydrothermal process	23.10	-	[53]
SPS with NaCl	29.30	24.90	[32]
SPS with protection layer	13.00	9.50	[17]
SPS	19.00	-	[28]
Powder injection molding	9.00	3.60	[54]
MASPS	27.10	7.70	Current study

## Data Availability

The corresponding author will provide the data used in this study upon reasonable request.
